# Trends of mitochondrial changes in AD: a bibliometric study

**DOI:** 10.3389/fnagi.2023.1136400

**Published:** 2023-05-16

**Authors:** Ruiyao Song, Yunchu Guo, Yu Fu, Hongling Ren, Hairong Wang, Hongting Yan, Yusong Ge

**Affiliations:** ^1^The Department of Neurology, The Second Hospital of Dalian Medical University, Dalian, China; ^2^The Department of Discipline Construction and Scientific Research Management, The Second Hospital of Dalian Medical University, Dalian, China

**Keywords:** Alzheimer’s disease, mitochondrial dysfunction, visualized analysis, bibliometric, VOSviewer

## Abstract

**Background:**

Alzheimer’s disease (AD) is a neurodegenerative disease characterized by progressive progress and memory loss, which eventually develops into dementia. It can cause personality disorders and decreased quality of life of patients. Currently, AD patients account for 60–70% of global dementia patients and the incidence rate of AD is increasing annually. AD not only causes pain to patients but also brings a heavy burden to the entire family. Studies have found that there is a connection between mitochondrial dysfunction and other biochemical changes in AD like classical neuropathological hallmarks (*β*-amyloid and tau protein), inflammation pathways, oxidative stress, and so on. Evidence shows that early treatment targeted directly to mitochondria could extend the lifespan of model mice and decrease the relevant neuropathological markers. Therefore, research on the mitochondrial dysfunction of AD can be of potential significance for clinical treatment. To date, few bibliometric analysis articles related to mitochondrial dysfunction of AD have been published. Bibliometric analysis refers to quantitatively analyzing certain aspects of articles like publishers, authors, and countries by using statistical and mathematical methods. Combined with statistical software, a large number of papers can be converted to visualization figures and tables, which provide vital information such as keyword hotspots and the names of contributing authors. Through the bibliometric analysis method, our study aimed to provide study trends and keyword hotpots for researchers to conduct further relevant research in this field.

**Methods:**

We used the Web of Science core collection database as a literature retrieval tool to obtain data related to mitochondrial changes in Alzheimer’s disease during the last 20 years. The retrieval type was [TS = (Alzheimer’s disease)] ND [TS = (mitochondrion)], ranging from January 1, 2000 to June 30, 2022. VOSviewer v1.6.18, Arcgis 10.8, and HistCite pro 2.1 were used to conduct data visualization analysis. VOSviewer v1.6.18 made relevant network visualization maps of the cooperative relationship between relevant countries, institutions, and authors (co-authorship), the frequency of different keywords appearing together (co-occurrence), and the frequency of different articles cited together (co-cited). Arcgis 10.8 created the world map of publications distribution in this field and Histcite pro 2.1 was used to count the local citation score (LCS) of references. In addition, Journal Citation Reports were used to consult the latest journal import factor and JCI quartile.

**Results:**

As of June 30, 2022, from the Web of Science core collection, we selected 2,474 original articles in English, excluding the document types of the news items, meeting abstracts, and some articles that had little relevance to our theme. The United States acted as the leader and enjoyed a high reputation in this field. The University of California System was the institution that made the greatest contribution (3.64% with 90 papers). Most articles were published in the Journal of Alzheimer’s Disease (8.21%, with 203 papers). The most frequently co-cited journal in Q1 was the Journal of Biological Chemistry (8,666 citations, TLS: 1039591). Russel H. Swerdlow (55 publications) was the most productive author and PH Reddy was the most co-cited author with 1,264 citations (TLS: 62971). The hotpots of mitochondrial dysfunction in AD were as follows: “oxidative stress,” “amyloid-beta-protein,” “tau,” “apoptosis,” “inflammation,” “autophagy,” “precursor protein,” “endoplasmic-reticulum,” “dynamics” and “mitochondrial unfolded protein response.”

**Conclusion:**

This bibliometric analysis research will help readers rapidly identify current hotpots and milestone studies related to directions of interest in AD research.

## 1. Introduction

AD is a neurodegenerative disease with a delitescence onset and progressive progression. Its core symptom is cognitive impairment, which gradually worsens over time, making it the leading cause of dementia in humans. According to the World Alzheimer Report, approximately 50 million people worldwide are suffering from dementia, of which 35 million are Alzheimer’s patients. According to data from different countries and regions, about 4.6% ~ 8.7% of adults over 60 years old are suffering from this disease and the incidence rate increases with age. The prevalence rate can be as high as 69.4% among people aged over 90. Because of its feature of progressive progress and gradually aggravated memory loss, AD patients will eventually completely lose their ability to take care of themselves. Their families need to spend enough time and money to take care of them. AD is a heavy burden and brings great pain to both patients and their families. Currently, clinical treatment drugs for AD do not effectively delay disease progression and it is, therefore, necessary to explore new drugs for this purpose.

At present researchers believe that the onset of AD is the result of a combination of multiple factors including amyloid plaques (Aβ plaque), neurofibrillary tangles (tau protein), vital mitochondrial dysfunction, oxidative stress, and inflammation pathways in brain neurons (Serý et al., 2013; Medala et al., 2021; Patro et al., 2021; Gowda et al., 2022). Neurocytes require more energy than other cells and mitochondria are important sites for cell energy metabolism. Studies have found decreased brain energy metabolism, increased oxidative stress, and active oxygen damage in AD neurons, which become evidence of mitochondrial dysfunction in AD (Frackowiak et al., 1981; Foster et al., 1983; Wang et al., 2006; Bonda et al., 2014). Early AD drugs concentrated on classical neuropathological changes including A*β* plaque and tau protein but had no expected therapeutic effect. Mitochondrial dysfunction not only leads to the abnormal energy metabolism of neurons but also interrelates with Aβ plaque, tau protein, aberrant reactive oxygen species(ROS) homeostasis, calcium overload, abnormal apoptosis, mitochondrial morphology, dynamics, DNA changes, and autophagy, etc. ROS is a natural by-product of normal oxygen metabolism. When ROS increases sharply, it can damage the cellular structure and cause oxidative stress (aberrant ROS homeostasis). (Wang et al., 2014; Varda et al., 2018; Judit and Russell, 2019). As the main production site of ROS, impaired mitochondria could lead to aberrant ROS homeostasis and oxidative stress. In healthy neurons, Ca^2+^ concentration is balanced at a lower level in the cytosol than in the extracellular space or certain intracellular compartments (Wang and Zheng, 2019). Mitochondria absorb Ca^2+^ at the expense of the electrochemical gradient generated during respiration. Mitochondrial Ca^2+^ overload means high uptake of mitochondrial Ca^2+^ which makes it easier to impair neuron functions and exacerbates AD progression by impairing mitochondrial respiration, increasing reactive oxygen species formation, inducing apoptosis, and damaging mitochondria recycling mitophagy (Wu et al., 2020). Mitochondria are essential energy generators for tissue homeostasis and channels for programmed apoptosis and necrotic cell death. The core functions require strict control of the quality and quantity of mitochondria. Mitochondrial autophagy (mitophagy) is the targeted phagocytosis and destruction of impaired mitochondria by cellular autophagy devices and is generally considered to be the main mechanism for mitochondrial quality control. Research shows that impaired mitophagy contributes to the accumulation of Aβ plaque and tau protein through oxidative stress and cellular energy deficits. These, in turn, can impair mitophagy (Kerr et al., 2017). In addition, mitochondrial dynamics showed a balanced process of mitochondrial fission and fusion. Evidence shows that the overexpression of dynamin-related protein1 (Drp1) interacts with other pathological changes and accelerates the disease progression (Chen and Chan, 2009). The interaction of multiple pathological changes contributes to the onset and progression of AD and mitochondrial dysfunction has a connection with other impact factors. Experimental drugs have targeted impaired mitochondria to the early onset of AD mice models and the drugs can extend their lifespan and decrease AD neuropathological hallmarks. Therefore, elucidating the correlation between mitochondrial dysfunction and it is thought that relevant drugs targeted at impaired mitochondria could be developed, providing potential treatment methods for AD (Judit and Russell, 2019).

Although many studies on mitochondria have been carried out, to date, no analyses have been conducted a systematic statistical review analysis. Many articles have been published related to mitochondrial dysfunction in AD, meaning it can be difficult for readers to quickly identify interesting articles. Bibliometric analysis refers to the use of mathematical and statistical methods to analyze all knowledge carriers quantitatively. The main measurement objects are literature volume, number of authors, institutions, countries, and keywords. VOSviewer v1.6.18, Arcgis 10.8, and HistCite pro 2.1 are used to analyze knowledge carriers and made visualization maps or tables. VOSviewer is a knowledge graph software based on thousands of articles that can construct relationships (like co-authorship, co-occurrence, and co-cited) between literature knowledge units (countries/regions, institutions, journals, authors, keywords) and achieve visual analysis through network maps. In the visualization network map, all nodes are divided into several clusters. Different clusters show different colors. The size of the nodes represents the frequency of occurrence of the specific knowledge unit and links between nodes represent the relevance between units. To view the publications of each country/region in the world more clearly, Arcgis 10.8 was used to create a world map of publications distribution. HistCite Pro 2.1 was used to analyze the LCS of references in this field so that we can quickly identify the influential articles on mitochondrial dysfunction of AD. By combining bibliometric analysis with statistical software, we can select a high number of publications from different countries/regions, institutions, journals, authors, the most highly-cited references, authors, journals, and popular keywords from thousands of papers published in recent decades.

Our study conducted a bibliometric analysis of published papers in the direction of mitochondrial dysfunction during the last 20 years and aimed to discover the current research hotspots and study trends to provide a roadmap for further research (Devos and Menard, 2019; Yuan et al., 2021; Ri et al., 2022).

## 2. Methods

The Web of Science core collection database was queried with the following search string: [TS = (Alzheimer’s disease)] ND [TS = (mitochondrion)]. The date ranged from January 1, 2000, to June 30, 2022. We excluded some special article types including meeting abstracts, news items, and so on. Articles, reviews, proceeding papers, and book chapters were collected. We also screened low-relevance papers in this field. Eventually, only the above four types of original articles written in English and published within the search range date were included.

We used the “export” function to save documents locally. The storage format of the document was as a “txt” file, which is suitable for VOSviewer v1.6.18 and HistCite pro 2.1 software. These data were imported into VOSviewer v 1.6.18 to create network visualization maps, aiming to analyze co-authorship, co-occurrence, and co-cited countries/regions, institutions, authors, journals, and hotspot keywords. In the visualization map, the size of the nodes represents the occurrence frequency of relevant elements, the thickness of the chain represents the degree of connection, and the color of the nodes indicates that they belong to different modules. Meanwhile, these data were imported into HistCite Pro 2.1 to analyze the LCS of articles. We made an Excel spreadsheet of the top 30 countries/regions publications and imported the table into Arcgis 10.8 to make a world map of publications distribution with a color gradient. The top 10 countries were marked on the world map. Journal Citation Reports (2021) was used to consult the latest journal import factor and JCI quartile.

## 3. Results

### 3.1. Publications

**Figure 1:**
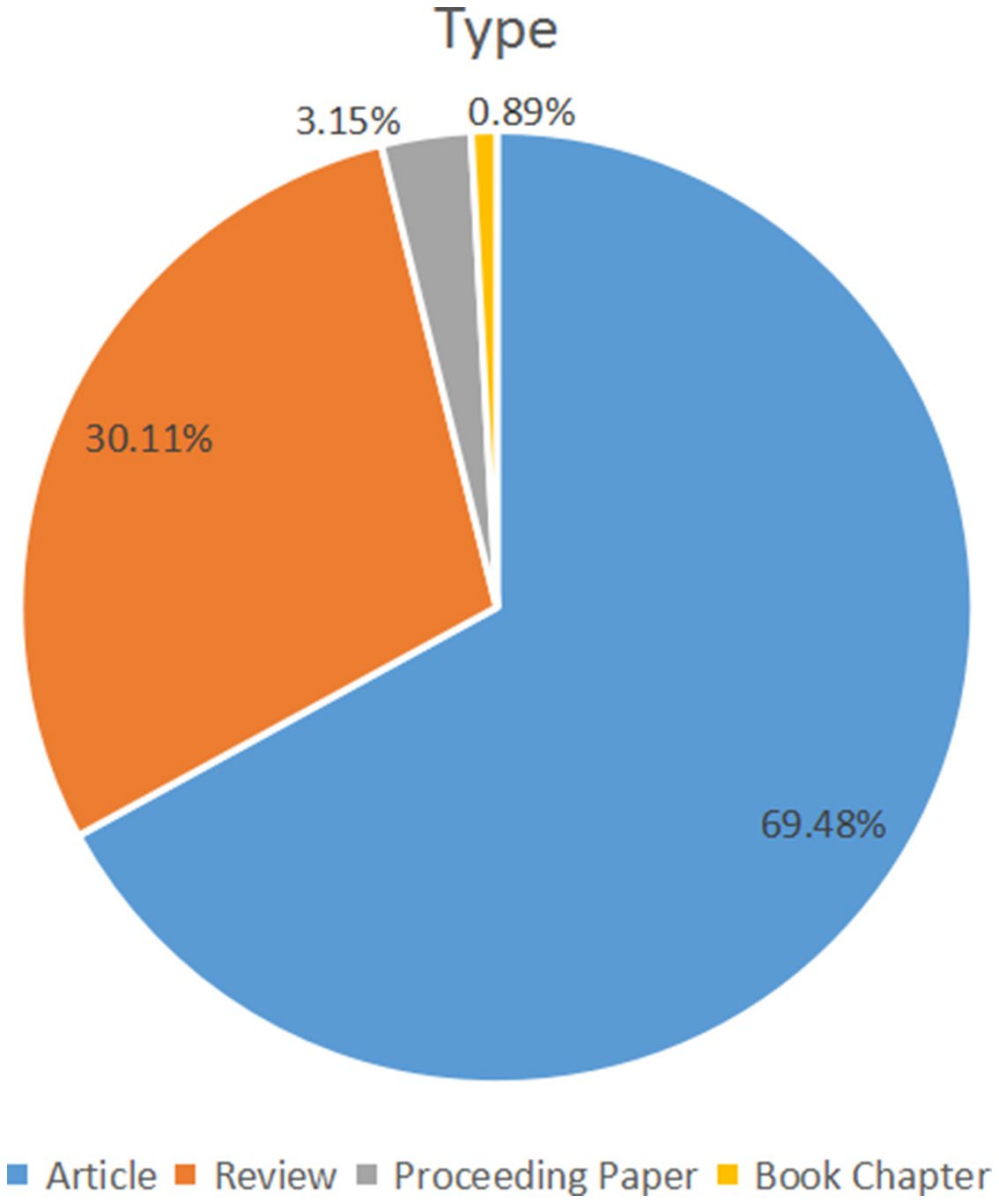
Study type composition summary in this field.

From January 1, 2000, to June 30, 2022, 2,474 papers were provided by the Web of Science core collection online database (2022.9), including 1719 original research articles (69.48%), 745 reviews (30.11%), 78 proceeding papers (3.15%), and 22 book chapters (0.89%). Other document types like meeting abstracts and news items were excluded (Figure 1). A total of 1,335 papers were open access.

**Figure 2:**
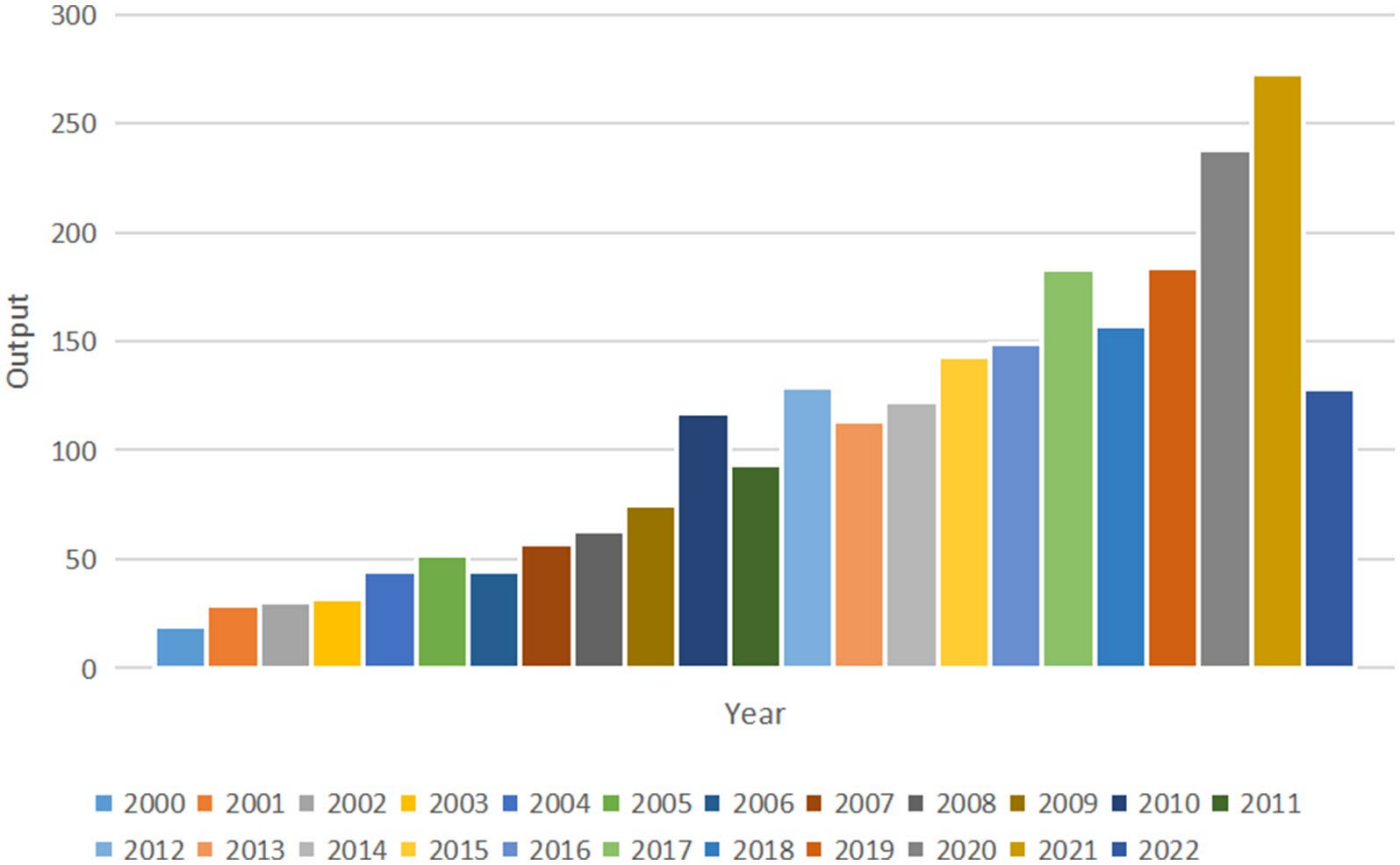
Total annual output from 2000 to 2022.

The annual publication output in this field with an overall growth trend is shown in Figure 2. The annual amount of research published exceeded 100 for the first time in 2010 (*n* = 117). More recently, 238 papers were published in 2020, 273 papers were published in 2021 and 128 papers were published in the first half of 2022. Compared with 20 years ago, the annual output in 2021 (*n* = 273) is nearly 15 times that of 2000 (*n* = 19).

### 3.2. Analysis of countries/regions, institutions, journals

**Figure 3:**
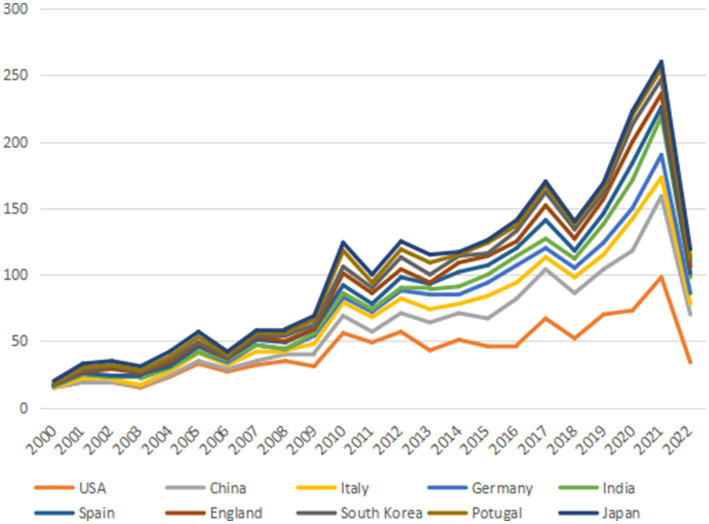
The annual publication output of the most 10 productive countries/regions.

**Figure 4:**
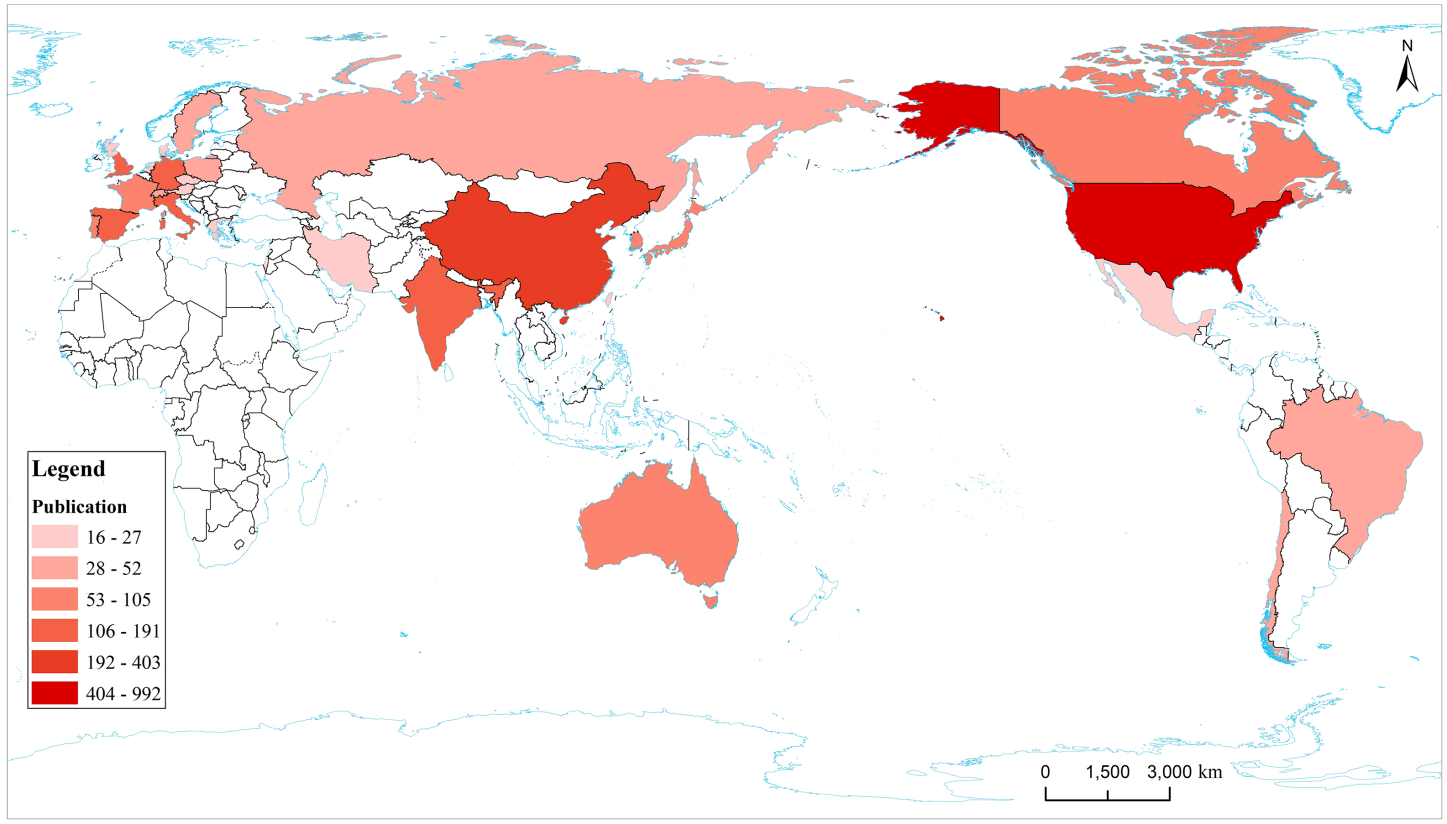
Country/region distribution map of the top 30 publications. The top 10 countries are marked on the map. The deeper color represents more publications.

**Figure 5:**
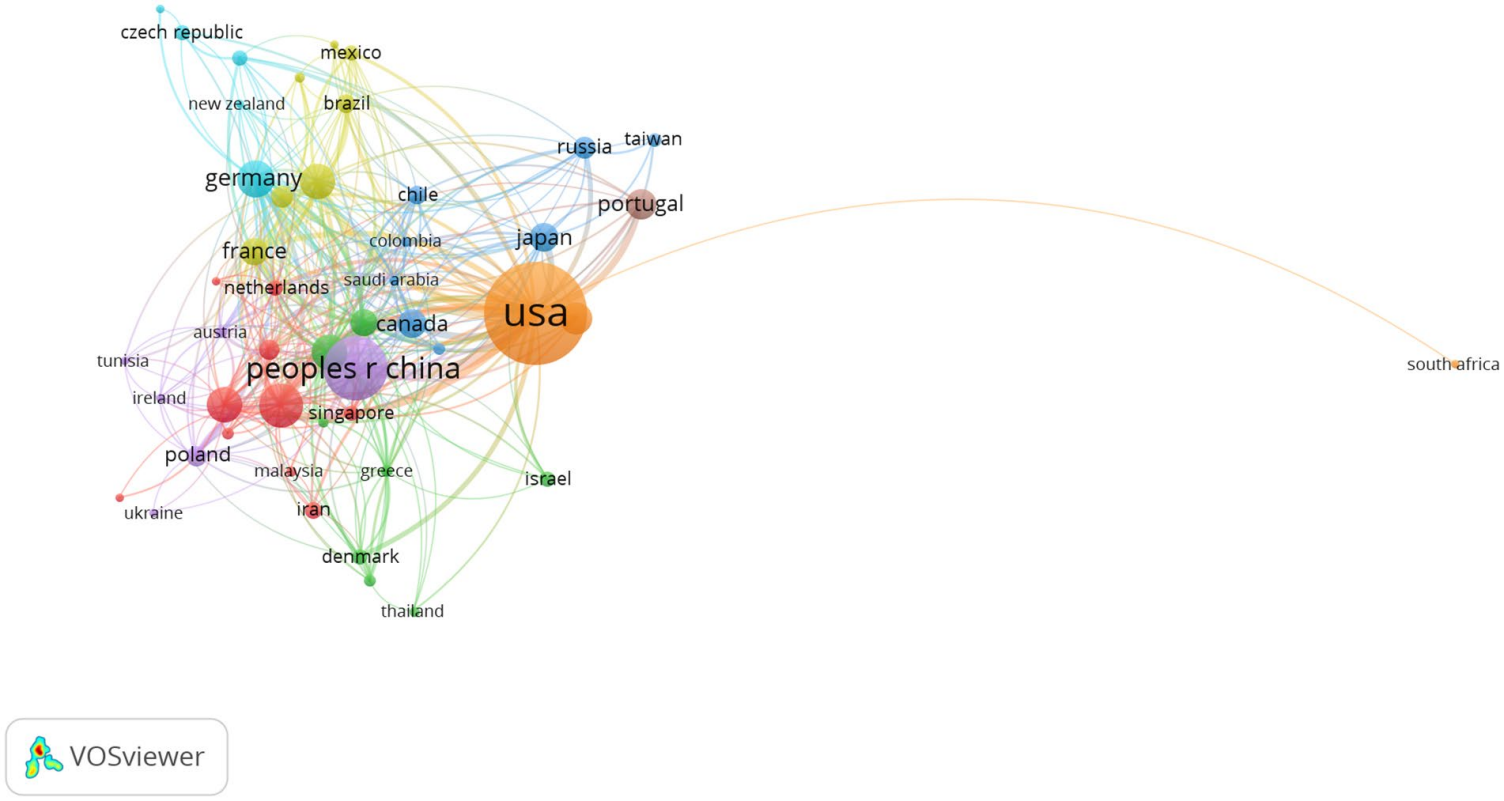
Network visualization map of co-authorship countries/regions. The nodes represent countries/regions and the larger the size of the node, the more publications from that country/regions. Nodes with the same color belong to the same cluster. The links between nodes represent the cooperation between countries/regions. The thickness of the links represents close cooperation between countries/regions.

The annual publication output from 2000 to 2022 in countries and regions is shown in Figure 3 and the distribution of publications in countries and regions worldwide is shown in Figure 4. The United States publishes the most papers every year. China’s publication output has grown annually since 2016 and its growth rate has increased significantly in that time, now ranking second. Table 1 shows the top 10 productive countries/regions in this field from 2000 to 2022. As mentioned, the most productive country is the United States (*n* = 992, 40.10%), followed by China (*n* = 403, 16.30%), Italy (*n* = 191, 7.72%), the Germany (*n* = 140, 5.66%), India (*n* = 124, 5.01%), Spain (*n* = 121, 4.89%), England (*n* = 120, 4.85%), South Korea (*n* = 105, 4.24%), Portugal (*n* = 95, 3.84%), and Japan (*n* = 84, 3.40%). In these countries/regions, the United States had 78,280 citations, 141 h-index, and its citations per publication (CPP) was 78.91. These all ranked first among 10 countries/regions. The network visualization map analysis of co-authorship of countries/regions (Figure 5) shows 48 countries/regions divided into seven clusters with different colors. The biggest node represented is the United States, which had the most publications (992 documents) and collaborators (43 countries/regions). Its total link strength (TLS) was 456. Some key partners of the United States included China, India, Italy, Germany, and Portugal.

**Table 1:** Top 10 productive countries/regions from 2000 to 2022.

Rank	Countries/regions	Records	Percentage (%)	H (%)-index	Citations	Citations per publication	Total link strength (TLS)
1	United States	992	40.10	141	78,280	78.91	456
2	China	403	16.30	66	12,898	32.00	118
3	Italy	191	7.72	63	11,928	62.45	117
4	Germany	140	5.66	44	7,098	50.70	125
5	India	124	5.01	58	4,703	37.93	73
6	Spain	121	4.89	57	6,052	50.02	110
7	England	120	4.85	41	8,610	71.75	123
8	South Korea	105	4.24	46	4,271	40.68	36
9	Portugal	95	3.84	41	5,322	56.02	43
10	Japan	84	3.40	42	6,243	74.32	61

**Figure 6:**
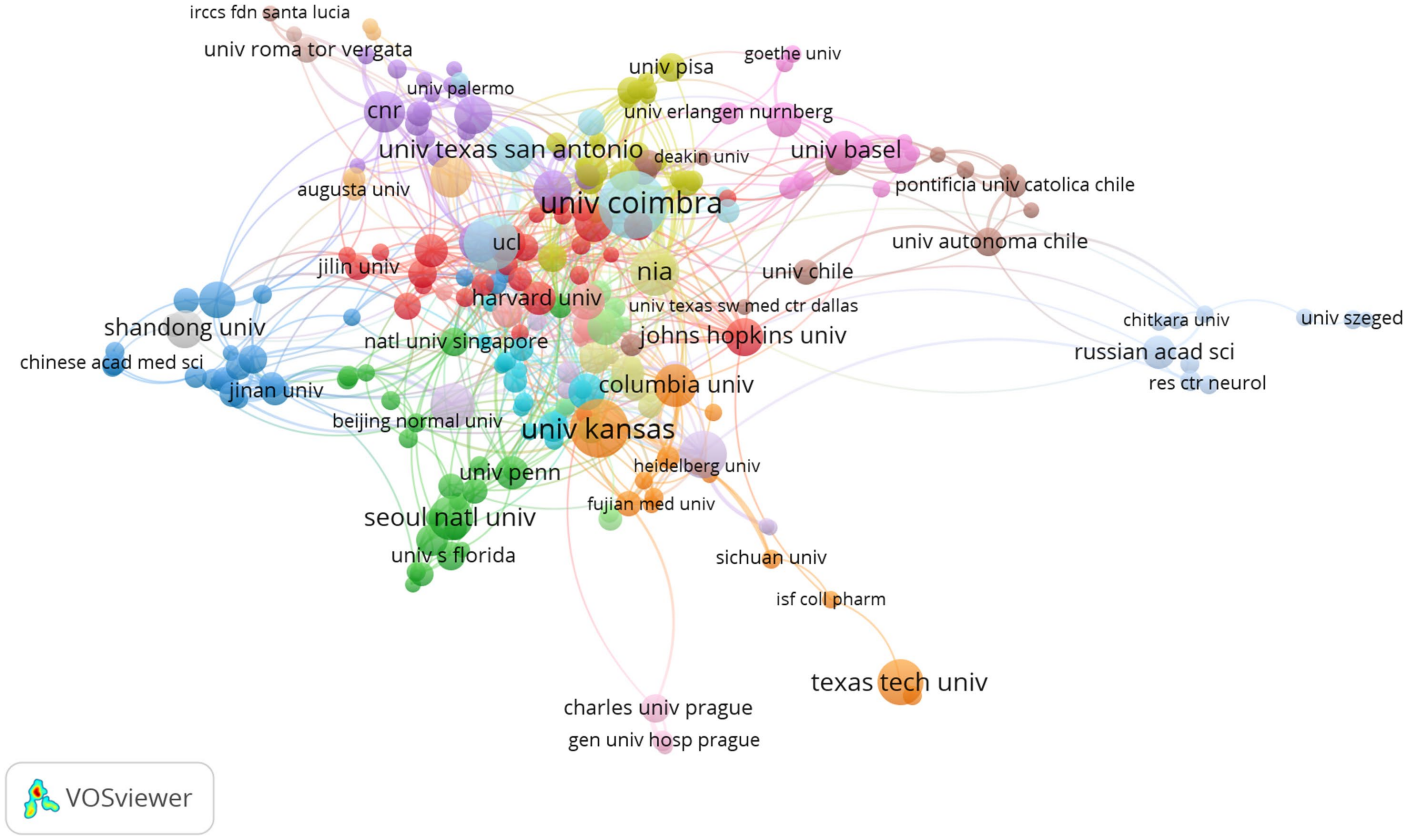
Network visualization map of co-authorship institutions. The nodes represent institutions and the larger the size of the node, the more publications from that institution. Nodes with the same color belong to the same cluster. The links between nodes represent the cooperation between institutions. The thickness of the links represents close cooperation between countries/regions.

The top 10 productive institutions are shown in Table 2. The institutions that made major contributions included: the University of California System (*n* = 90, 3.64%), Universidade DE Comibra (*n* = 78, 3.15%), University of Texas System (*n* = 77, 3.11%), Cornell University (*n* = 62, 2.51%), University of Kansas (*n* = 62, 2.51%). The University of California System had the highest H (%)-index (50) and Case Western Reserve University was the institution with the most citations per publication (135.30). Seven of the top 10 institutions were from the United States and others were from Portugal, Britain, and France. The network visualization map of co-authorship of institutions (Figure 6) showed that a total of 249 institutions were divided into 19 clusters because of their different degrees of correlation (links: 827, total link strength: 1292) and some institutions had no cooperation with others and were excluded. Links between institutions represented their cooperations in this field. The largest node referred to Universidade DE Coimbra (publications: 78, TLS: 48), which had some main collaborators with the University of Kansas, Case Western Reserve University, Columbia University, University of Texas at San Antonio, and University of Virginia.

**Table 2:** The top 10 productive institutions from 2000 to 2022.

Rank	Institutions	Records	Percentage (%)	H (%) -index	Citations per publication	Location
1	University of California System	90	3.64	50	85.50	The United States
2	Universidade DE Comibra	78	3.15	42	60.45	Portugal
3	University of Texas System	77	3.11	42	90.79	The United States
4	Cornell University	62	2.51	37	132.71	The United States
5	University of Kansas	62	2.51	28	70.60	The United States
6	National Institutes of Health NIH USA	61	2.47	38	111.72	The United States
7	Case Western Reserve University	54	2.18	37	135.30	The United States
8	University of London	51	2.06	31	77.82	England
9	Harvard University	48	1.94	26	52.94	The United States
10	Udice French Research Universities	48	1.94	24	54.48	France

**Figure 7:**
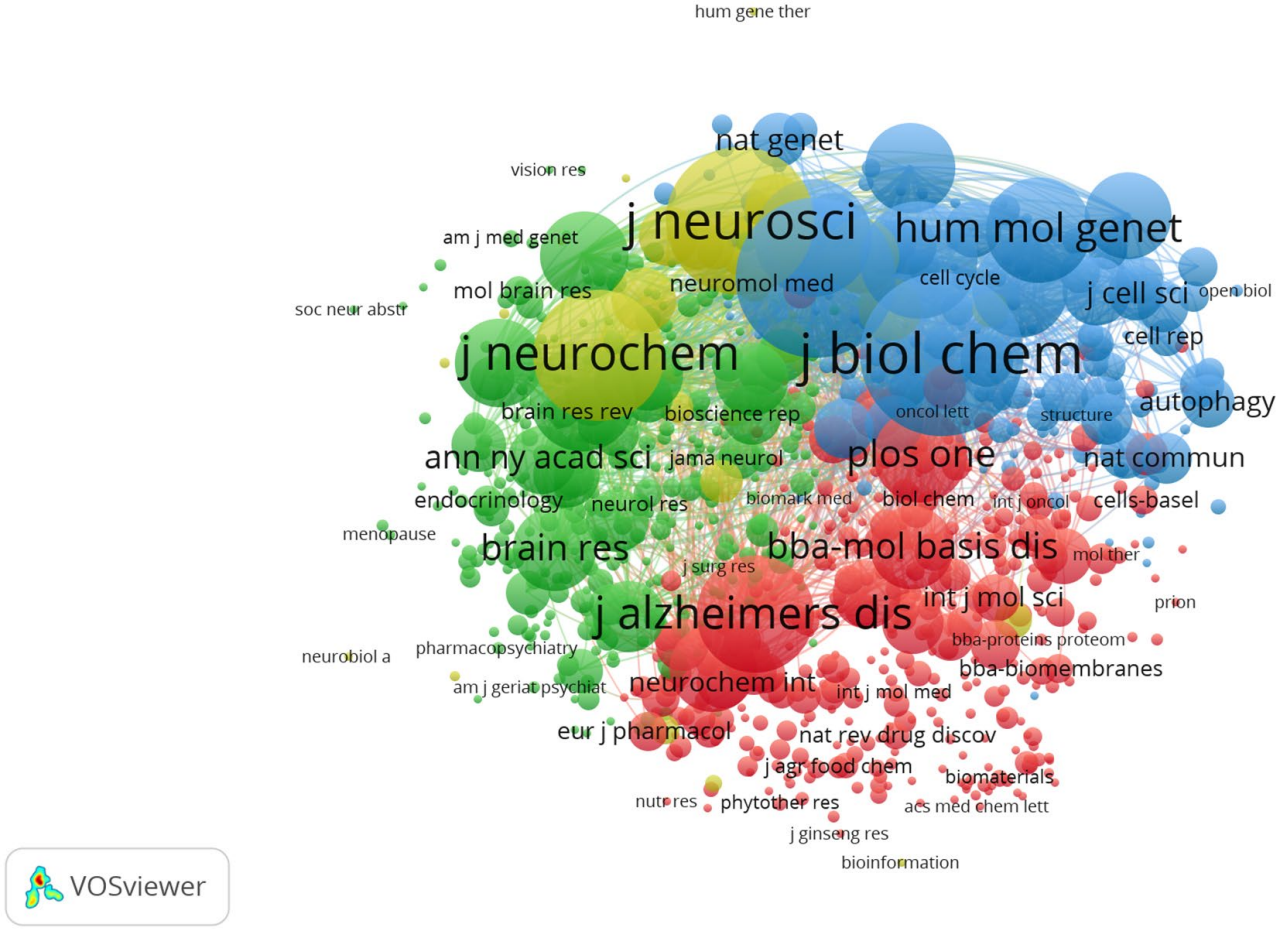
Network visualization map of co-cited journals. The nodes represent journals and the larger the size of the node, the more citations of journals. The links between nodes represent the co-cited relationship between journals. The thickness of links represents the frequency of the co-cited relationship between journals.

The top 10 productive journals related to mitochondrial dysfunction of Alzheimer’s disease are shown in Table 3. The top 10 productive journals published 597 articles, accounting for 24.13%, nearly one-fifth of the total, and most were classified in Q2. Table 3 indicated that the Journal of Alzheimer’s Disease (*n* = 203, 8.21%, Q2) published the most research in this field and had the highest H-index (61) followed by Molecular Neurobiology (*n* = 69, 2.79%), Neurobiology of Aging (n = 49, 1.98%), the International Journal of Molecular Sciences (*n* = 46, 1.86%), and the Journal of Neurochemistry (*n* = 44, 1.78%). Among these journals, Cells (*n* = 35, 1.41%, Q2) ranked tenth and had the highest IF (7.666) and its CCP was 15.49. Biochimica ET Biophysica ACTA-Molecular Basis of Disease (*n* = 50, 1.36%, Q1) had the highest CCP of 138.12 and had the second highest IF (6.633). The analysis diagram of co-cited journals showed in Figure 7 and Table 4. From Table 4, The most frequently co-cited journal was the Journal of Biological Chemistry (citations: 8666 TLS: 1039591). Among these journals, the Proceedings of The National Academy of Sciences of The United States of America had the highest IF of 12.779 and was classified in Q1. Figure 7 shows a network visualization map of co-cited journals. A total of 877 items were divided into four groups and the representative journal in each group included: Journal of Alzheimer’s Disease (in cluster1 shown in red), Neurobiology of Aging (in cluster 2 shown in green), the Proceedings of The National Academy of Sciences of The United States of America (in cluster 3 shown in blue), and the Journal of Neuroscience (in cluster 4 shown in yellow).

**Table 3:** The top 10 productive journals from 2000 to 2022.

Rank	Journals	Records	Percentage (%)	IF	H (%)-index	Citations per publication	Quartile in category
1	Journal of Alzheimer’s Disease	203	8.21	4.160	61	54.53	Q2
2	Molecular Neurobiology	69	2.79	5.686	28	38.80	Q2
3	Neurobiology of Aging	49	1.98	5.133	30	59.29	Q2
4	International Journal of Molecular Sciences	46	1.86	6.208	18	19.02	Q1
5	Journal of Neurochemistry	44	1.78	5.546	31	95.91	Q2
6	Biochimica ET Biophysica ACTA-Molecular Basis of Disease	42	1.70	6.633	32	138.12	Q1
7	PloS One	37	1.50	3.752	23	56.81	Q2
8	Mitochondrion	36	1.46	4.534	18	40.56	Q3
8	Neurochemical Research	36	1.46	4.414	20	43.36	Q2
10	Cells	35	1.41	7.666	10	15.49	Q2

**Table 4:** The top 10 co-cited journals from 2000 to 2022.

Rank	Journals	Citations	Total link strength	IF	H (%)-index	Quartile in category
1	Journal of Biological Chemistry	8,666	1,039,591	5.485	57	Q1
2	Proceedings of The National Academy of Sciences of The United States of America	8,311	1,052,362	12.779	14	Q1
3	Journal of Neuroscience	7,084	860,667	6.709	31	Q1
4	Journal of neurochemistry	5,649	699,116	5.546	29	Q2
5	Journal of Alzheimer’s Disease	4,533	565,283	4.160	21	Q2
6	Nature	4,516	588,446	69.504	1	Q1
7	Science	4,454	561,845	63.832	29	Q2
8	Neurobiology of Aging	3,687	465,324	5.686	34	Q2
9	Human Molecular Genetics	3,668	511,788	5.133	33	Q2
10	Cells	2,920	384,107	7.666	1	Q2

### 3.3. Analysis of productive authors and co-cited authors

**Figure 8:**
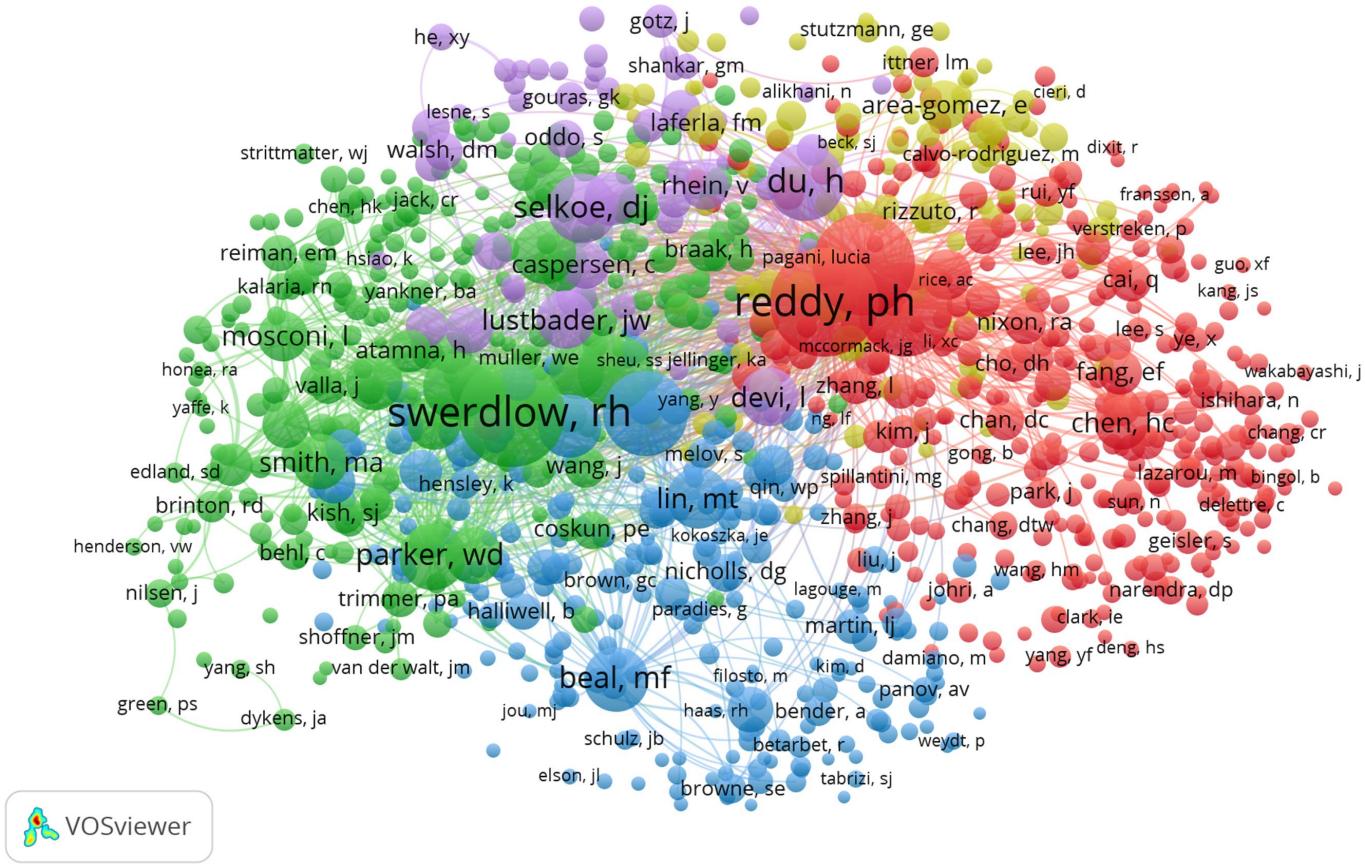
Network visualization map of co-cited authors. The nodes represent authors and the larger the size of the node, the more citations of authors. The links between nodes represent the co-cited relationship between authors. The thickness of links represents the frequency of the co-cited relationship between authors.

Table 5 describes the top 10 productive authors who published research relevant research. Russell H. Swerdlow was the most productive author in this field with 55 articles, accounting for 2.22%. The analysis of co-cited authors was shown in Figure 8 and Table 6. The most frequently co-cited author was P. H. Reddy (citations: 1264, TLS: 62971) followed by R. H. Swerdlow (citations: 1221, TLS: 67444), M. Manczak (citations: 1118, TLS: 59789), X. L. Wang (citations: 970, TLS: 53343) and M. P. Mattson (citations: 772, 34,088).

**Table 5:** The top 10 productive authors in this field from 2000 to 2022.

Rank	Authors	Records	Percentage (%)
1	Swerdlow, Russell H.	55	2.22
2	Moreira, Paula	42	1.70
3	Perry, George	39	1.58
4	Reddy, P. Hemachandra (USA)	33	1.33
5	Eckert, Anne	33	1.33
6	Reddy, P. Hemachandra (India)	24	0.97
7	Yan, Shirley ShiDu	24	0.97
8	Brinto, Roberta D.	22	0.89
9	Moreira, Paula I.	20	0.80
10	Gibson, Gary E.	19	0.77

**Table 6:** The top 10 co-cited authors in this field from 2000 to 2022.

Rank	Co-cited authors	Citations	Total link strength
1	Reddy, PH	1,264	62,971
2	Swerdlow, RH	1,221	67,444
3	Manczak, M	1,118	59,789
4	Wang, XL	970	53,343
5	Mattson, MP	772	34,088
6	Du, H	652	31,703
7	Moreira, PI	613	32,215
8	Gibson, GE	485	24,891
9	Parker, WD	467	29,544
10	Beal, MF	464	24,121

### 3.4. Analysis of high-cited references and co-cited references

**Figure 9:**
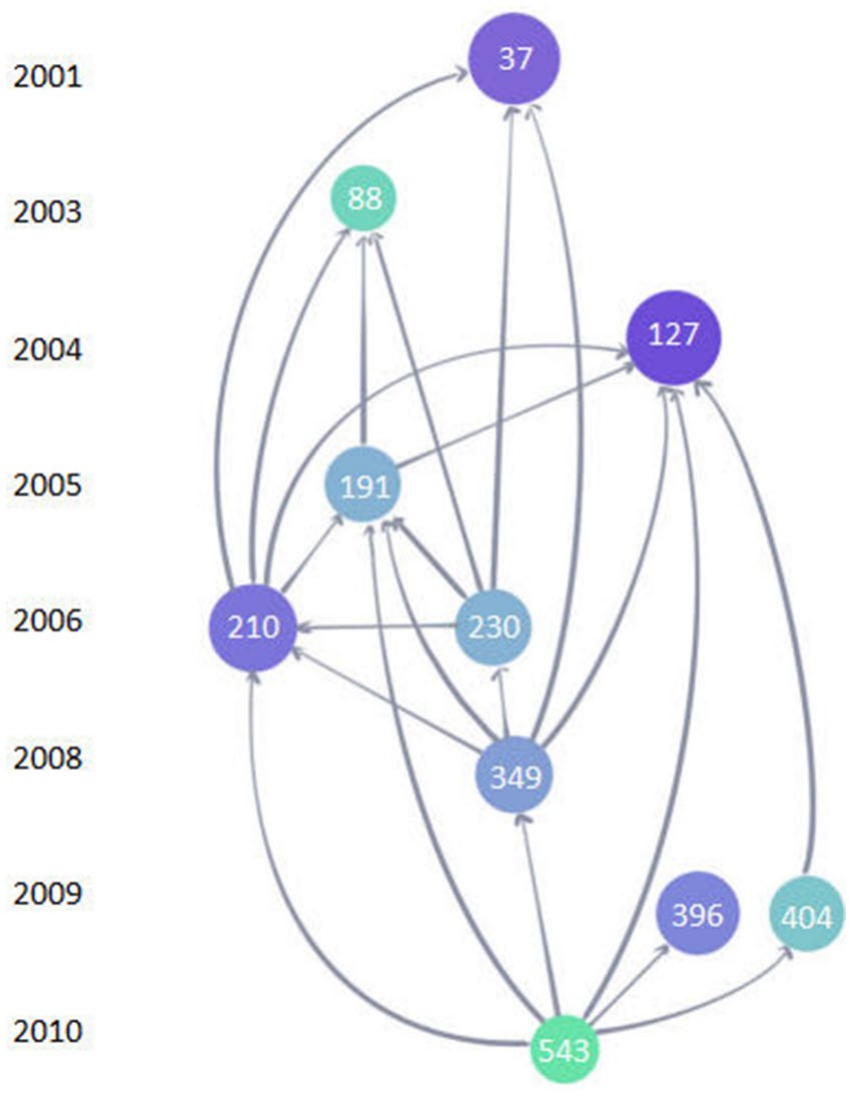
The top 10 LCS map of references. Each circle represents a reference. A larger circle with a deeper color of blue means a higher LCS. The number in the circle represents the ordinal number of the article in the Web of Science core collection database. The links between circles represent a citation relationship between articles. The object pointed to by the arrow is the cited reference.

**Figure 10:**
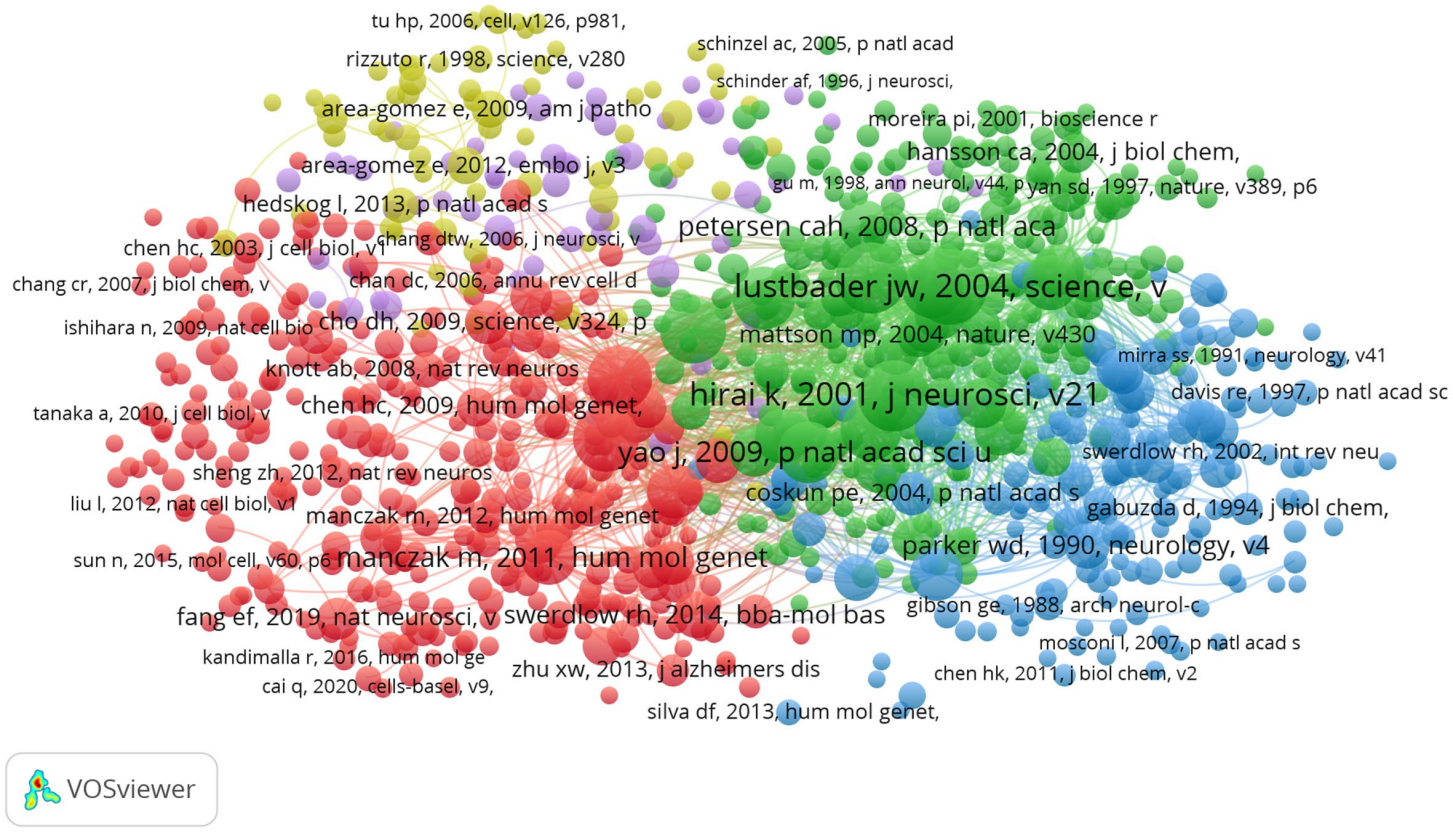
Network visualization map of co-cited references. The nodes represent references and the larger the size of the node, the more citations of references. Items in the same cluster belong to the same research direction. The links between nodes represent the co-cited relationship between references. The thickness of links represents the frequency of the co-cited relationship between references.

The top 10 LCS of articles is shown in Figure 9. The most frequently cited reference with a high reputation was “ABAD directly links A beta to mitochondrial toxicity in Alzheimer’s disease” produced by J. W. Lustbader, published in Science (No.127, IF = 6.709, Q1) with the LCS of 363. This article demonstrated that A beta-binding alcohol dehydrogenase (ABAD) is a direct molecular link from Abeta to mitochondrial toxicity by experimenting with the transgenic mice overexpressing ABAD in an A beta-rich. Therefore, it suggested that the ABAD-A beta interaction may be a therapeutic target in AD (Lustbader et al., 2004). Figure 10 shows the network visualization map of co-cited references in this field with 887 items divided into five clusters. Studies on mitochondrial dysfunction of AD are mainly devoted to the following: Abnormal mitochondrial dynamics (Wang et al., 2009), the direct relationship between abnormal mitochondria and oxidative damage (Cardoso et al., 2004; Devi et al., 2006), the cytochrome C oxidase deficiency (Parker et al., 1990), modulation of the endoplasmic reticulum-mitochondria interface and high levels of mitochondrial DNA deletions in substantia nigra neurons (De Brito and Scorrano, 2008; Louise et al., 2013; Swerdlow et al., 2014).

### 3.5. Analysis of keywords

**Figure 11:**
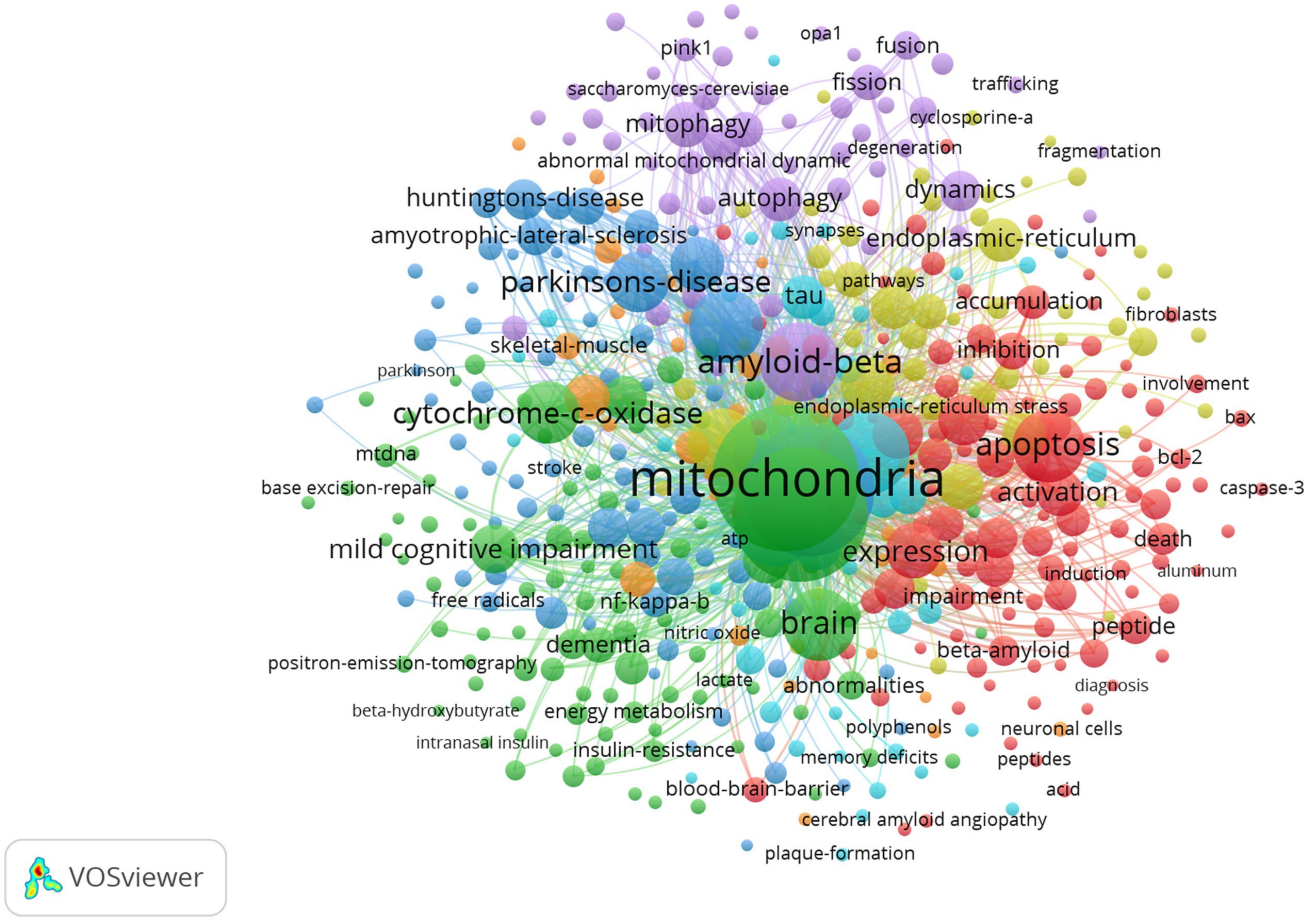
Network visualization map of cluster analysis of co-occurrence keywords. Items in the same cluster belong to the same research direction in this field. The larger the size of the node, the higher the frequency of the keyword. The links between keywords represent a co-occurrence relationship.

**Figure 12:**
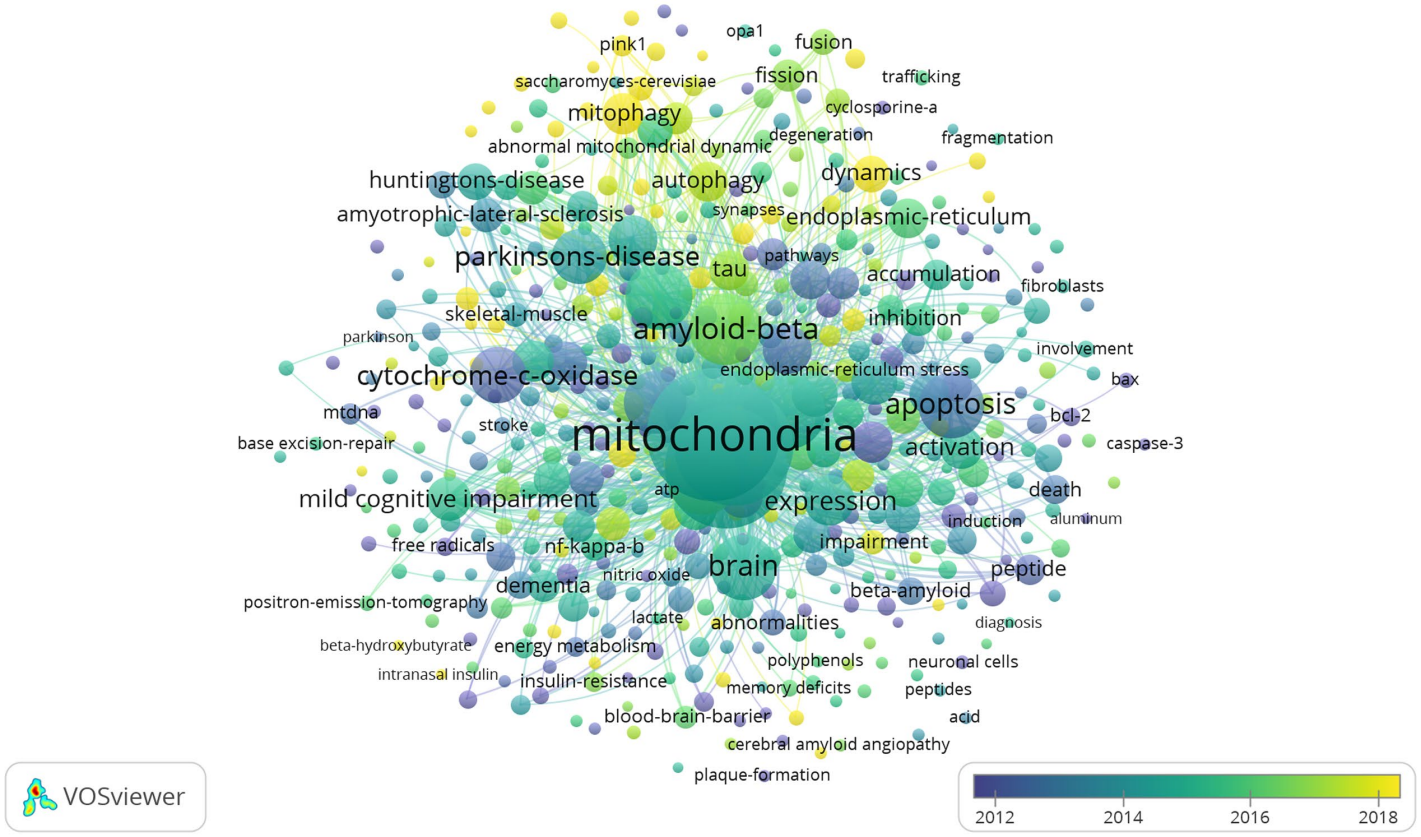
Network visualization map of timing analysis of co-occurrence keywords from 2012 to 2022. The closer the color of the node is to yellow, the later the occurrence of keywords.

The network visualization map of co-occurrence keywords shown in Figure 11 (cluster analysis) and Figure 12 (timing analysis) includes 478 items divided into seven clusters and 21,833 links. The minimum number of occurrences of a keyword was 10. The repeated and meaningless keywords were eliminated from the map. Analyzing the frequency of different groups of keywords provides a convenient approach for us to identify hot topics in the mitochondrial study field of AD, which are classified into clusters representing various subtopics in our study field. According to Figure 11, which shows the cluster analysis of keyword co-occurrence, the most popular keywords related to mitochondrial dysfunction in AD included “oxidative stress,” “amyloid beta,” “amyloid precursor protein,” “cytochrome-c-oxidase,” and “autophagy.” Reading the Tenth figure (timing analysis of keyword co-occurrence), indicates that “calcium homeostasis,” “precursor protein,” “amyloid beta,” “autophagy,” “dynamics,” “abnormal interaction,” and “unfolded protein response (UPR)” were popular hot spots in order of time (from 2012). Critical research related to these hotspots is further illustrated in the discussion.

## 4. Discussion

As a neurodegenerative disease, AD is characterized by progressive progress and memory loss that eventually contribute to dementia, which causes patients to lose their ability to take care of themselves, with most of them dying of infection eventually. During this period, a large amount of time and money is spent, creating a heavy burden on the patient’s family. Due to these facts, finding approaches to delaying disease progression and increasing the quality of a patient’s life is key to AD research. AD is considered a multifactorial disease, including Aβ plaque, tau protein, inflammation pathways, mitochondrial dysfunction, and oxidative stress. Additionally, several risk factors such as aging, genetic factors, head injuries, vascular diseases, infections, and environmental factors play a role in the disease (Breijyeh and Karaman, 2020). Because of the unideal therapeutic effect based on the classical neuropathological hallmarks of A*β* plaques and tau protein, we concentrated on articles related to mitochondrion changes in neuron cells, which is one of the research directions of AD. Through the bibliometric analysis method, we aimed to illustrate keyword hotspots and research trends in this field that are convenient for researchers in conducting further studies. We used the Web of Science core collection database to search relevant articles from January 1, 2000, to June 30, 2022. Some special types of articles such as meeting abstracts, editorial materials, and new items were excluded. Through screening articles with low relevance, a total of 2,474 original papers in English were collected. Figure 2 shows an overall growth trend annually indicating there are increasing studies on AD.

From the distribution of countries/regions, the United States is the main driving force, with a high academic reputation in the field of AD. In just over 20 years, the United States published 992 articles, approximately 40% of the total, with 78,280 citations, 141 H-index, and 78.91 CCP, all ranking first among all countries/regions. Furthermore, 43 countries/regions had a close cooperative relationship with the United States, such as China, Japan, Portugal, and France. China ranked second (403 articles) with quick progress in these years. Other countries/regions like Italy, Germany, India, Spain, England, South Korea, Portugal, and Japan all had a lower publication amount (*n* < 200) compared with the United States. Despite all this, these countries/regions still play an important role and promote the development of this field.

Our analysis of institutions indicated that most of them were based in the United States, which again confirmed its significant scientific influence. The University of California System had the highest record of 90, accounting for 3.64% and its 50 H-index also ranked first among all institutions. Case Western Reserve University had the highest CCP of 135.30, which demonstrated that it published more high-quality articles with great contributions.

Analyzing the top 10 productive journals, we know that the Journal of Alzheimer’s Disease published the most articles, 203 in this field, Biochimica ET Biophysica ACTA-Molecular Basis of Disease, ranking sixth, had the highest CCP of 138.12, and Cells had the highest IF at 7.666, ranked tenth. All of them were vital information sources. In addition, the Journal of Biological Chemistry ranked first among institutions with the highest citation of 8,666, showing its great influence on AD research.

Russell H. Swerdlow was the most productive author with 55 records. Their most cited research, “The Alzheimer’s disease mitochondrial cascade hypothesis: Progress and Perspective,” discusses the mitochondrial cascade hypothesis in the context of recent AD biomarker studies, diagnostic criteria, and clinical trials and offers a unique perspective into what sporadic, late-onset AD is and how to best treat it (Swerdlow et al., 2014; Swerdlow, 2018). P.H. Reddy was the most popular co-cited author with 1,264 citations. Their research named “Amyloid Beta and Phosphorylated Tau-Induced Defective Autophagy and Mitophagy in Alzheimer’s Disease” highlights recent development of A and P-Tau-induced defective autophagy and mitophagy in AD and summarizes several aspects of mitochondrial dysfunction, including abnormal mitochondrial dynamics, defective mitochondrial biogenesis, reduced ATP, increased free radicals and lipid peroxidation, and decreased COX activity and calcium dyshomeostasis in AD pathogenesis (Reddy and Oliver, 2019). These articles provide reliable information in this academic field.

The top 10 highest LCS of references in this field can be summarized into the following aspects: the relationship between oxidative stress and mitochondria, mitochondrial dynamics, the influence of Aβ plaque on mitochondria, and some special molecular treatment targets. The article with the highest LCS named “ABAD directly links A-beta to mitochondrial toxicity in Alzheimer’s disease” illustrated that ABAD was the intermediate protein molecule that Aβ caused mitochondrial toxicity. This made ABAD possible to be an available therapeutic target (Lustbader et al., 2004). As a disease of aging, the study showed that AD had high levels of oxidative stress compared with elderly controls because of an imbalance of production and elimination of ROS (Wang et al., 2014). This could lead to damaged mitochondria which would aggravate oxidative stress. The interaction between oxidative stress and mitochondria injury and a series of complicated changes might cause neuron degeneration (Emerit et al., 2004; Beal, 2005). In the top 10 highest cited references, mitochondrial dynamics was another hotspot, including fission, fusion, transport, and autophagy, which involved mitochondrial critical subcellular compartments, content exchange, shape control, communication with cytosol, and quality control. Mutation of relevant proteins would contribute to the abnormal distribution of mitochondria and then lead to neurocytopathy (Chen and Chan, 2009; Wang et al., 2009). Aβ plaque is a critical pathological change of AD that could elevate the expression of mitochondrial genes like an increase of hydrogen peroxide and cytochrome c oxidase (Manczak et al., 2006). Mitochondria played a critical role in the development and plasticity of synaptic. To prevent synaptic changes caused by Aβ, the overexpression of mitochondrial dynamic protein contributed to an imbalance in dynamics like the production of SNO-Drp1, which was the combination of A*β* and NO (an important biological messenger in the brain) (Cho et al., 2009). Relevant dynamic changes could therefore be a potential novel therapeutic strategy for treating AD (Wang et al., 2009).

We analyzed clusters of popular keywords including “oxidative stress,” “amyloid-beta-protein,” “tau,” “apoptosis,” “inflammation,” “autophagy,” “precursor protein,” “endoplasmic-reticulum,” “dynamics” and “mitochondrial unfolded protein response.” The present review has discussed some recent hotspots in this field, providing researchers with more research directions. The studies on AD discussed here are all based on its basic pathological changes (A*β* plaque and tau protein), which interact with mitochondrial dysfunctions including abnormal mitochondria dynamics, altered mitochondrial morphology, altered mitochondrial gene expression, increased free radical production, lipid peroxidation, reduced COX activity, and reduced ATP production. Studies have shown that Aβ/tau protein can impair mitochondrial quality control systems including autophagy and mitophagy, and eventually lead to the accumulation of abnormal mitochondria (Maria et al., 2006). Mitophagy, a selective form of autophagy in mitochondria, constitutes a key pathway of mitochondrial quality control mechanisms involving the sequestration of defective mitochondria into autophagosomes for subsequent lysosomal degradation. “Mitochondrial Quality Control and Disease: Insights into Ischemia–Reperfusion Injury,” a highly cited review published by A. R. Anzell in 2017, provides a synopsis of the molecular mechanisms involved in the quality control mechanism regulated by mitochondrial dynamics and mitophagy, which could help us to speculate novel therapeutic interventions of AD (Anzell et al., 2017). The review of “Mitophagy in Alzheimer’s Disease and Other Age-Related Neurodegenerative Disease” summarized several mechanisms of mitophagy defects in AD, including mitophagy *in vivo*, neuronal mitophagy, and lipid-mediated mitophagy. These molecular mechanisms were thought to be critical factors in aging and neurodegeneration in recent years (Cai and Jeong, 2020). Eshraghi briefly discussed an overview of autophagy and mitophagy in AD and then provided a comprehensive discussion on the role of these pathways in microglia and their involvement in AD pathogenesis (Eshraghi et al., 2021). Mitophagy was considered the next hope for finding a viable therapeutic target in AD. Thus, by analyzing the map, we can speculate that “mitochondrial dynamics” and “mitophagy” will continue to be a research trend in the next few years. Researchers can concentrate on these molecular mechanisms for further study.

In terms of the oxidative stress of AD, in addition to the above classic articles, recent studies have concentrated on chronic inflammation and abnormal brain metabolism induced by type 2 diabetes mellitus (T2DM). T2DM leads to body metabolic disorders that activate oxidative stress, abnormal protein processing, and proinflammatory cytokines, causing chronic inflammation and impaired neuronal insulin signaling in the brain (Kandimalla et al., 2017). The increasing inflammatory mediators could cross the blood–brain barrier and cause brain insulin resistance followed by inducing ROS and proinflammatory cytokines (Cameron and Landreth, 2010). Aβ oligomers could activate inflammatory mediators, which trigger neuroinflammation and cause over-activation of N-methyl-D-aspartate (NMDA) receptors, which are viewed as a critical factor in the excessive production of ROS followed by excessive calcium ion-induced mitochondrial dysfunction (Go et al., 2016). Conversely, these proinflammatory cytokines would inhibit phagocytosis thus enhancing Aβ accumulation, which causes the development of AD (Cai et al., 2016; Zhang et al., 2019). Therefore, further study is required to establish how neuronal insulin signaling can be stimulated, how oxidative stress and inflammatory effects can be prevented, and approaches to restoring mitochondrial dysfunction and insulin sensibility (Shen et al., 2022). In addition, as a neurodegenerative disease, AD exhibits extensive oxidative stress throughout the body and structurally and functionally damaged mitochondria are also vulnerable to oxidative stress. The interaction between them contributes to the initiation and/or amplification of reactive oxygen species that are critical to the pathogenesis of AD (Wang et al., 2014; Chen and Zhong, 2019; Ionescu-Tucker and Cotman, 2021). Neurons affected in AD experience mitochondrial dysfunction and a bioenergetic deficit that occurs early and promotes the disease-defining amyloid beta peptide and Tau pathologies (Kerr et al., 2017). These pathological changes also instigate an abnormal apoptotic cascade in susceptible brain regions. The apoptotic players in these regions affect cellular organelles (mitochondria and endoplasmic reticulum) and interact with trophic factors, and several signaling pathways (Sharma et al., 2021). Meanwhile, a significant change of cox enzyme could be an important molecular component of the mechanisms underlying AD (Morais et al., 2021; Xu et al., 2021).

As another recent research hotspot, mitochondrial unfolded protein response (UPRmt) belonging to the mitochondrial quality control system is a mitochondria stress response. UPRmt is the transcriptional activation program of mitochondrial chaperone proteins and proteases are initiated to maintain proteostasis in mitochondria. Mitochondrial chaperone proteins help misfolded proteins to restore to normal conformation and newly synthesized proteins to fold correctly. Proteases help to degrade useless proteins. Aβ and tau deposition contribute to the accumulation of damaged or unfolded protein, which will increase the expression levels of mitochondrial chaperone proteins and proteases (Li et al., 2020). Meanwhile, AD pathological changes also contribute to the endoplasmic reticulum (ER) stress, which caused the build-up of unfolded or misfolded protein with the ER, disturbing the ER and cellular homeostasis, devoting it to the onset and development of AD. Adaptive UPR signaling is activated to reverse mild ER stress and regain homeostasis, thereby preventing exacerbation (Yang et al., 2015; Ren et al., 2021). However, irreversible ER stress and excessive UPR activation can contribute to neuroinflammation and neuronal cell death, which is associated with the advanced stage of AD pathogenesis (Amir et al., 2022). Therefore, future research should examine to “what extent ER stress is reversible in neurons,” how other factors determine between adaptive and maladaptive UPR signaling in either the production or death of neurons in AD, how to alter damaged UPRmt, and what components of maladaptive UPR can be targeted can be effective to alleviate AD pathophysiology.

In general, researchers have concentrated on undertaking basic medicine studies of AD. However, most of the time, clinical treatment with relevant theories has not achieved good results due to the onset complexity of AD. There is still a long way for researchers to go on exploring the neuropathology of AD.

Previous bibliometric studies of AD have analyzed the relationship between the intestine and AD, epilepsy and AD, and brain energy metabolism disorder in AD (Du et al., 2021; Trejo-Castro et al., 2022; Zhang et al., 2022). Our study aimed to collect articles related to mitochondrial dysfunction in AD and analyzed them quantitatively and qualitatively, which no one has done yet. Meanwhile, we searched articles from the Web of Science core collection database so the extracted data are reliable, comprehensive, and highly recognized (Fei et al., 2022).

### 4.1. Limitations

Our study also has some limitations as we could not retrieve all articles meeting the requirements of this field. Due to not all types of papers were selected, our findings might not be fair to authors who published papers in excluded types. We may ignore their contributions in this field. Our study also only included articles in the first half of 2022, meaning these data do not represent the whole research trend for that year. Finally, our analysis was subjective and it can’t represent all people’s views.

## 5. Conclusion

The highest number of publications were from the United States followed by China, Italy, Germany, and India. The primary partners of the United States were China, Italy, Germany, India, and Portugal.

The University of California System in the United States and Russell. H. Swerdlow were the most cited institution and author, respectively. The Journal of Alzheimer’s Disease was the highest-cited journal.

The research hotspot in this field included the following five aspects: abnormal mitochondrial dynamics, the direct relationship between abnormal mitochondria and oxidative damage, the cytochrome C oxidase deficiency, modulation of the endoplasmic reticulum-mitochondria interface, and high levels of mitochondrial DNA deletions in substantia nigra neurons.

The research trend from 2012 to 2021 was: “calcium homeostasis,” “precursor protein,” “amyloid beta,” “autophagy,” “dynamics,” “abnormal interaction,” and “unfolded protein response (UPR).”

This bibliometric analysis study aimed to help researchers and non-researchers quickly find research hotspots and landmark studies related to mitochondrial dysfunction in AD. There are shortcomings from insufficient data or analysis and more progressive studies are needed in the future.

## Data availability statement

The original contributions presented in the study are included in the article/supplementary material, further inquiries can be directed to the corresponding author.

## Author contributions

YGe conceptualized this study and designed this project. RS and HY undertook data curation. RS performed the major procedures and wrote the manuscript. YGe revised the manuscript and approved the final manuscript. YGu, YF, HR, and HW assisted in the analysis of the data.

## Funding

This study was funded by the Natural Science Foundation of Liaoning Province [2022-MS-317], the United Fund of the Second Hospital of Dalian Medical University and Dalian Institute of Chemical Physics, Chinese Academy of Sciences [UF-ZD-202012], and the “1 + X” program for Clinical Competency enhancement–Improvement of Clinical Technology Project, The Second Hospital of Dalian Medical University [2022LCJSGC04].

## Conflict of interest

The author declares that the research was conducted in the absence of any commercial or financial relationships that could be construed as a potential conflict of interest.

## Publisher’s note

All claims expressed in this article are solely those of the authors and do not necessarily represent those of their affiliated organizations, or those of the publisher, the editors and the reviewers. Any product that may be evaluated in this article, or claim that may be made by its manufacturer, is not guaranteed or endorsed by the publisher.
